# Supplementary material for: Strength, extent and duration of secondary hyperalgesia induced by high-frequency electrical stimulation of the foot compared to the volar forearm of healthy human volunteers
Source: PLoS One. 2025 Feb 7;20(2):e0318934. doi: 10.1371/journal.pone.0318934 (PMC11805354; doi:10.1371/journal.pone.0318934)
Supplement: Attachment — Submitted filename: Rebutal Letter PLOSONE Lebrun.docx [file pone.0318934.s001.docx]

To the editor,

Please find hereby each reviewer’s comments addressed.

*Reviewer #1: Thank you for the opportunity to review this well-written manuscript. The setup seems complex, as do some aspects of the statistical analysis.*

*We thank the Reviewer for this comment. In the revised manuscript, we have tried to further streamline the presentation of the statistical analyses (page 8 lines 171-172; 190-192, 202-204).

Was the protocol of the study published in advance? If so, where can it be accessed? Information on this should be included in the manuscript.*
*We thank the Reviewer for this comment. In this exploratory study, planned comparisons were defined before data collection, but the protocol was not published in advance. Only the decision to compare the time courses of the HFS-induced secondary hyperalgesia using an exponential decay function was taken a posteriori, after visualization of overall time courses across testing sites. This has now been clarified in the manuscript (page 5 lines 86-87).*

*Was there statistical planning to determine the number of subjects needed?*

*We thank the Reviewer for this comment. This was an exploratory study rather than a confirmatory study. Effect sizes were not known. Our sample size of 32 participants (16 per group) was similar to the size of the samples used in several previous studies assessing differences in HFS-induced secondary mechanical hyperalgesia such as differences related to the frequency of stimulation (15 and 16 participants in van den Broeke et al., J Neurophysiol 2019) or differences related to the pattern of HFS stimulation (15 participants in Gousset et al., J Neurophysiol 2020).*

*This has now been clarified in the manuscript (page 5 lines 92-97). Effect size estimates of our key outcomes are reported in the manuscript.

Lines 141-143: What was the frequency of the applied stimuli? You state that you never used the same location twice, however wind-up could also occur if stimuli are applied in a quick repetitive execution within a small area.*

*We thank the Reviewer for this comment. Stimuli were applied every 5 seconds. This information has now been added to the manuscript (page 7, line 150).*

*Of note, we propose here a table, showing paired t-test proving that repetitive application of pinprick did not significantly change perceived intensity at group level.*

| ***Paired Samples T-Test*** | | | | | | | | | | | |
| --- | --- | --- | --- | --- | --- | --- | --- | --- | --- | --- | --- |
| ***Measure 1*** | |  | | ***Measure 2*** | | ***t*** | | ***df*** | | ***p*** | |
| *HFS side 1st stim* |  | *-* |  | *HFS side 2nd stim* |  | *-1.027* |  | *31* |  | *0.312* |  |
| *HFS side 1st stim* |  | *-* |  | *HFS side 3rd stim* |  | *-0.145* |  | *31* |  | *0.886* |  |
| *HFS side 2nd stim* |  | *-* |  | *HFS side 3rd stim* |  | *0.778* |  | *31* |  | *0.443* |  |
| *Control side 1st stim* |  | *-* |  | *Control side 2nd stim* |  | *-1.743* |  | *31* |  | *0.091* |  |
| *Control side 1st stim* |  | *-* |  | *Control side 3rd stim* |  | *-0.679* |  | *31* |  | *0.502* |  |
| *Control side 2nd stim* |  | *-* |  | *Control side 3rd stim* |  | *0.790* |  | *31* |  | *0.435* |  |
|  | | | | | | | | | | | |
| *Note.  Student's t-test.* | | | | | | | | | | | |

*eg. Lines 255-257 Reporting of the statistical data should probably always note if IQR or CI is provided.*

*We thank the Reviewer for this comment. This is now specified throughout the manuscript (lines 220, 249, 250, 275, 283, 291).*

*Lines 305-310 I think this is too speculative ("thus"), your study does not seem to be designed to draw this conclusion.
We agree with the Reviewer. The word “thus” was replaced by “might” line 329.*

*Figure 1 shows spellcheck highlighting ("HFS-sensitized limb", Tbaseline)*

*We thank the Reviewer for spotting this mistake which was corrected.*

*mult. Figures area radius is shown in mm, discussion within the article mentions cm. Same units would look more homogenous.*

*We agree with the Reviewer. Area radius is now reported in mm throughout the manuscript.*

*Fig 3b, Fig 4d should show the different meaning of the dotted line within the image.*

*This information has now been added.*

*Reviewer #2: The sample size was very poor. It should be increased the sample size to conclude this kind of research work. Although the research work is technically sound, and presented in an intelligible fashion.*

*We thank the Reviewer for this comment. This was an exploratory study rather than a confirmatory study. Effect sizes were not known. Our sample size of 32 participants (16 per group) was similar to the size of the samples used in several previous studies assessing differences in HFS-induced secondary mechanical hyperalgesia such as differences related to the frequency of stimulation (15 and 16 participants in van den Broeke et al., J Neurophysiol 2019) or differences related to the pattern of HFS stimulation (15 participants in Gousset et al., J Neurophysiol 2020).*

*This has now been clarified in the manuscript (page 5 lines 92-97). Effect size estimates of our key outcomes are reported in the manuscript.*
